# Evaluating PubMed-Indexed Publications of Applicants Successfully Matching into the Top 50 Urology Residency Programs in the 2021-2023 Cycles

**DOI:** 10.7759/cureus.37996

**Published:** 2023-04-22

**Authors:** Sahaam Mirza, Tatum Williamson, Moben Mirza, Robert D Arnce

**Affiliations:** 1 Medicine, Kansas City University of Medicine and Biosciences, Kansas City, USA; 2 Urology, Beth Israel Deaconess Medical Center, Harvard Medical School, Boston, USA; 3 Urology, University of Kansas Health System, Kansas City, USA; 4 College of Osteopathic Medicine, Kansas City University of Medicine and Biosciences, Joplin, USA

**Keywords:** pub-med indexed research, urology, research productivity, urology education, urology residency

## Abstract

Background and objective

Urology residency match occurs through the American Urological Association (AUA), and hence information about the success of applicants in finding a match is not readily available. The average number of publications a successful urology applicant has when applying for residency is unknown. In light of this, we conducted this study to examine the number of PubMed-indexed research projects involving US senior medical students who successfully matched into the top 50 urology residency programs in the 2021, 2022, and 2023 match cycles. We also assessed these applicants based on their medical schools and gender.

Methods

Doximity Residency Navigator was used to generate the top 50 residency programs as sorted by reputation. Newly matched residents were found using program Twitter accounts and residency program websites. PubMed was queried for peer-reviewed publications of incoming interns.

Results

The average number of publications across all incoming interns in the three years was 3.65. The average number of urology-specific publications was 1.86 and that of first-author urology publications was 1.11. The median number of total publications for matched applicants was 2, and applicants with a total of five publications were in the 75th percentile for research productivity.

Conclusion

A successful applicant had two PubMed-indexed urology papers on average and also had a urology-specific first-author paper in the cycles we surveyed. There has been an increase in publications per applicant when comparing the results to previous application cycles, which can be attributed to post-pandemic changes.

## Introduction

Urology has become one of the most competitive medical specialties, with a match rate that has fluctuated between 70 and 80% for graduating senior medical students in the United States in the past three years. While desirable applicant qualifications vary among programs, program directors among all specialties consistently rank The United States Medical Licensing Exam (USMLE) scores, honors in clinical rotations, strong letters of recommendation, and research experience as the most important factors when deciding which applicants to interview [1]. For most specialties, applicants are able to gauge their competitiveness through the National Residency Matching Program’s (NRMP) Charting Outcomes in the Match reports, which provide annual statistics regarding board scores, volunteer experiences, and research projects. However, since urology does not participate in the NRMP, no central database is available for urology applicants to gauge their competitiveness for residency applications. Hence, urology applicants must rely on other sources to determine their chances of success in the match.

A recent study showed that since the outbreak of the coronavirus disease 2019 (COVID-19) pandemic, the most important factors in terms of applicant assessments have been letters of recommendation on rotations, urology clerkship grades, research experience, and visa status of the applicant [2]. Warren et al. (2020) have quantified the PubMed-indexed research productivity of medical students who matched at the top 50 urology residency programs from 2017 to 2020 [3]. Since then, applicants have had to face the challenges of the COVID-19 pandemic, which led to the complete cancellation of audition rotations for the 2020 residency application cycle and limitations being imposed on rotations for the 2021 cycle. Audition rotations have historically represented an important way for applicants to establish relationships with residency programs and the limitations imposed on these rotations may have required program directors to distinguish applicants using other factors [4]. USMLE Step 1 exam has now shifted to a simple pass/fail grading structure, which may have led to an increase in research productivity as applicants look to strengthen other aspects of their application. The lack of numeric scores will push programs to adopt a holistic evaluation approach, with a greater focus on research output.

In this study, we strove to quantify the PubMed-indexed research productivity of successful urology applicants at the top 50 urology residencies in the last three application cycles. We expected to find an increase in the average number of PubMed-indexed publications in comparison to the results of Warren et al. (2020).

## Materials and methods

The Doximity Residency Navigator program was used to generate the top 50 urology residency programs in the US for each year as sorted by reputation, based on objective data and surveys completed by urologists. This program has been used in similar publications and is widely regarded as an effective tool by applicants when applying to residency programs. To obtain the names of incoming interns, we used publicly available Twitter posts, an Excel spreadsheet, and individual websites of urology residencies. The mentioned spreadsheet includes the name of individuals and the program they successfully matched to and was completed anonymously. The spreadsheet was then verified with information on Twitter posts and the websites of programs. Based on verification and corroboration between the three sources, we were able to compile the names of the incoming interns at the top 50 urology programs.

PubMed was queried pertaining to data for March 2021, 2022, and 2023 regarding all forms of PubMed-indexed research productivity for the incoming urology interns of each year. All peer-reviewed publications that had been published by March of the respective application cycle for each year were included in the study. Individuals with generic names were cross-referenced on Twitter, Linkedin, and Google as there would be publications from multiple institutions. However, the profile of 28 individuals could not be verified due to the huge amount of publications that were under their specific names. Ten international medical graduates (IMGs) were excluded from the data analysis because they had completed research fellowships. We also collected the following demographic data: gender, medical school, urology-specific first-author publications, urology-specific publications, and total publications. The top 10 medical schools that had the highest number of successful applicants were grouped together and labeled as “feeder schools”.

The collected data was then analyzed by using RStudio (Version 1.14.1106). The data were then compared between the three application cycles and found to have non-normally distributed results. Our findings were then compared to those captured by Warren et al. [3]. The study received IRB approval from Freeman Health System, Joplin, MO.

## Results

We captured data on 468 matched applicants across the top 50 urology programs for the 2021-2023 cycles by using Doximity Residency Navigator. Most programs ranked in the top 50 for all three years but the lists were not identical.

The average number of publications across all incoming interns in the three cycles was 3.65 (SD: 4.56). Furthermore, the average number of urology-specific publications was 1.86 (SD: 3.29), and that of the first-author urology-specific publications was 1.1 (SD: 2.09) (Table 1). The median number of total publications for successfully matched applicants was 2 and those in the top 75th percentile had a total of five publications. Furthermore, 57.3% of matched applications had at least one urology-related publication, 46.8% had at least one first-author urology-related publication, and 77.7% had at least one publication in the medical field. The majority did not have more than two first-author or any urology-related publications. An uptrend in publications was noted when compared to the findings of Warren et al. (2020). In our data, we observed a trend similar to the findings of Warren et al. as the number of PubMed-indexed publications decreased as residency program ranking decreased.

**Table 1:** Data on the number of PubMed-indexed research publications for the incoming intern classes of 2021, 2022, and 2023 IQR: interquartile range; SD: standard deviation

	2021, average (SD)	2022, average (SD)	2023, average (SD)	2021-2023, average SD	2021, median (IQR)	2022, median (IQR)	2023, median (IQR)	2021-2023, median (IQR)
Overall publications	3.83 (4.82)	4.3 (5.11)	2.94 (3.68)	3.65 (4.56)	2 (4)	3 (3.75)	1 (4)	2 (4)
Urology-related publications	2.38 (2.64)	1.78 (2.78)	1.58 (2.77)	1.86 (3.29)	1 (2.75)	1 (2.75)	1 (2)	1 (2)
First-author urology publications	1.04 (2.01)	1.21 (2.00)	1.11 (2.22)	1.11 (2.09)	1 (1)	0.5 (2)	0 (1)	0 (1)

The findings of Warren et al. were as follows - overall: 2.38 ± 4.19; urology: 1.05 ± 3.19; first-author: 0.80 ± 1.77.

In our study, women made up 41.6%, 38.3%, and 45.5% of the incoming urology interns in 2021, 2022, and 2023 cycles respectively. The average number of first-author urology-specific, urology-specific, and total publications that were PubMed-indexed for women in the three application cycles were 0.91, 1.49, and 3.19 respectively.

Lastly, 10 feeder medical schools accounted for 26.5%, 22.2%, and 25.4% of the incoming urology interns at the top 50 urology programs for 2021, 2022, and 2023 cycles respectively (Tables 2-4). The average first-author urology-specific, urology-specific, and total publications that were PubMed-indexed from the 10 feeder medical schools in the three application cycles were 1.47, 2.73, and 4.32 respectively. Applicants from these medical schools showed an increase in research productivity when compared to others.

**Table 2:** List of feeder schools representing 26.5% of incoming interns for the class of 2020-21

Schools (2020-21)	Number of successful applicants at the top 50 programs
University of California, Los Angeles	6
University of Maryland	5
SUNY Downstate	4
Tufts University	4
University of Southern California	4
Icahn School of Medicine	4
University of Iowa	4
Northwestern University	4
University of Pennsylvania	4
University of Michigan	4

**Table 3:** List of feeder schools representing 22.2% of incoming interns for the class of 2021-22

Schools (2021-22)	Number of successful applicants at the top 50 programs
University of Texas Southwestern	6
Medical College of Georgia	4
University of Florida	4
University of Pennsylvania	4
University of Michigan	4
Brown University	3
Cornell University	3
Northwestern University	3
University of California, San Francisco	3
University of Nebraska	3

**Table 4:** List of feeder schools representing 25.4% of incoming interns for the class of 2022-23

Schools (2022-23)	Number of successful applicants at the top 50 programs
University of California, Los Angeles	7
Medical College of Wisconsin	4
University of Michigan	4
Northwestern University	4
University of Indiana	4
Vanderbilt University	4
University of California, San Diego	3
Harvard	3
Johns Hopkins	3
Rutgers RWJ	3

## Discussion

The current study aimed to quantify the number of PubMed-indexed research publications by incoming urology interns at the 50 top-ranked urology residency programs and to find other characteristics that may be associated with a greater chance of matching. This research project was conducted with reference to the findings of Warren et al. in the hopes of building on their results and looking for other characteristics of successful urology applicants, especially in the context of the onset of COVID-19 and its accompanying effects on medical education.

Our work examined the characteristics of 468 successfully matched applicants at the top 50 urology residency programs in the last three years. There was a cumulative increase in total publications and first-author urology-specific research from the 2021 to 2022 cycle. Data for the 2023 cycle showed a decrease in all domains of research. This could be due to the pandemic; most students complete research during their second and third year of medical school, which was when COVID-19 was at its peak for the 2023 applicants. Disruption in research infrastructure, along with general medical education, due to the pandemic could be the reason for such a decrease [5]. There was an increase in all publication categories when compared to the findings of Warren et al. This highlights how there has been an increase in research productivity among those who were able to match into the top 50 urology residency programs.

It is also important to note that there were applicants in the past three years who were able to successfully match without having any urology-specific research that was PubMed-indexed, as there were 124 applicants who were able to match in these three cycles. However, Faber et al. found that the majority of urology residency programs (98%) that responded to their survey required participation in scholarly activity in order for residents to graduate in urology [6]. It is advisable for medical students to learn the basics of research prior to beginning their residency.

Other characteristics measured by our study included gender, degree, and medical school of applicants. Women had fewer PubMed-indexed publications across all types in comparison to their male counterparts. This finding could potentially be attributed to the barriers women face in the field of urology and the difficulties in finding research opportunities. Velez et al. found that there are institutional policies and pervasive biases that create such barriers for women [7]. Furthermore, researchers have found that there has been an increase in female representation in urological publications; however, their papers are less likely to be cited and still make up a smaller proportion of urological literature [8]. Women represented 33.61%, 35.61%, and 37.1% of applicants who matched successfully into all urology programs in the years 2021, 2022, and 2023 respectively. The proportion of women within the top 50 programs was higher in comparison to all urology programs, and it is a positive sign to see an increase in women applicants being accepted into top urology programs. However, there is still room for improvement as women are underrepresented in the field of urology as a whole [9].

Although IMGs were excluded from data analysis due to their research fellowships already being completed, we found that on average they had 24.4 publications that were PubMed-indexed, which shows the need for stronger application credentials given the lower match rate for IMGs. Mignucci-Jiménez et al. showed that the research productivity of IMGs is considered superior to US graduates in the field of neurosurgery [10]. More research publications strengthen an applicant's overall application credentials, which is necessary for IMGs as they need to overcome barriers such as visa requirements.

Lastly, 10 medical schools accounted for over 20% of successful applications at the top 50 urology programs in each of the last three application cycles. Successful applicants from these feeder schools had a greater number of PubMed-indexed research across all types of research. Individuals who attend these institutions have a greater chance to publish high-profile research as many of these schools were among the top 10 National Institutes of Health (NIH)-funded medical schools in the relevant years. Colaco et al. found that urology faculty members who were funded by the NIH had higher research productivity than those who were not funded by the NIH [11]. Therefore, students who attend medical schools that receive greater funding from the NIH will have the opportunity to work with faculty members with increased research productivity.

With Step 1’s transformed grading system, the medical school an individual attends can play a major role in their success in matching into a top urology program. These medical schools provide students with unique resources that can give them an advantage in becoming competitive applicants, such as networking opportunities and higher exposure to the field itself. Kutikov et al. highlighted in their study the importance of strong mentorship and cited it as a major factor as to why certain medical schools send more students into urology residencies than others [12]. Furthermore, many students are not exposed to urology until their clinical clerkship years. Researchers have found that medical students' interest in urology increased after completing urological hands-on training [13]. Certain medical schools provide opportunities in urology prior to clinical clerkship years, which could explain why they have more successful urology applicants.

The increase in publications when compared to the findings of Warren et al. can be attributed to multiple potential factors such as an increase in competitiveness and the onset of COVID-19 [3]. As the number of applicants applying to urology is growing faster than the residency positions available, the field is becoming more competitive. Furthermore, there is an increase in variability in the applicant pool from year to year. From 2020 to 2023, there has been an increase of 32 positions offered, which is outweighed by 67 applicants registering for the American Urological Association (AUA) match. Applicants have differentiated themselves from one another through research publications, especially in urology, which displays their dedication to the field [14]. Furthermore, the increase in PubMed-indexed research can be attributed to the emergence of COVID-19, which has placed more pressure on applicants to differentiate themselves. Virtual interviews have challenged applicants to stand out more and made it difficult to assess program culture to see how they would fit within it [15].

There are several limitations to our study. Firstly, it did not take into account the fact that some applicants may have published under different names and also did not specify which applicants may have taken time off from medical school to pursue research opportunities. Furthermore, data from four programs were excluded due to a lack of information available online at the time of data collection. Next, many applicants pursuing competitive specialties choose to take a year off to pursue research opportunities to bolster their application credentials to match into programs that are research-heavy. Therefore, these applicants may have a higher research output than those who graduated within four years. Also, both sets of data were collected in March of each respective year, which was well beyond the Electronic Residency Application Service (ERAS) submission date. Thus, many of the research projects indexed in PubMed by the date of our search may not have been completed by the submission deadline. However, applicants would still likely have had these projects listed on their applications as “submitted” and would have still been able to discuss these projects during interviews. Lastly, another limitation involves the definition of a “top 50 program”. This can be a subjective ranking as different lists use different metrics. For instance, this study would have different results if we used the top 50 programs based on NIH urology funding.

## Conclusions

Research productivity remains a valuable asset on any graduating medical school student’s residency application. The majority of applicants who successfully matched into a top 50 urology program had at least one first-author urology-specific PubMed-indexed publication, and those matching at topmost programs had more. Urology-specific research, especially first-author urology papers, can improve an applicant’s success at matching into a top program.

As urology is not a required rotation at most medical schools, many applicants usually decide on a career in urology late in their medical school trajectory and are thus unable to complete urology-specific research before the application deadline. Applicants in this position can still be successful as program directors now tend to perform a holistic review. However, with the changed grading system of Step 1, there may be a greater emphasis on research productivity in the future.
